# Recent advances in modified starch based biodegradable food packaging: A review

**DOI:** 10.1016/j.heliyon.2024.e27453

**Published:** 2024-03-08

**Authors:** Saeeda Fatima, Muhammad Rehan Khan, Imran Ahmad, Muhammad Bilal Sadiq

**Affiliations:** a Kauser Abdulla Malik School of Life Sciences, Forman Christian College (A Chartered University), Lahore, 54600, Pakistan; b Department of Agricultural Science, University of Naples Federico II, Via Università 133, 80055, Portici, NA, Italy; c Food Agriculture and Biotechnology Innovation Lab (FABIL), Florida International University, Biscayne Bay Campus, North Miami, Florida, USA

**Keywords:** Modified starch, Biodegradable films, Food packaging, Resistant starch, Native starch, Biopolymer

## Abstract

This study reviews the importance of resistant starch (RS) as the polymer of choice for biodegradable food packaging and highlights the RS types and modification methods for developing RS from native starch (NS). NS is used in packaging because of its vast availability, low cost and film forming capacity. However, application of starch is restricted due to its high moisture sensitivity and hydrophilic nature. The modification of NS into RS improves the film forming characteristics and extends the applications of starch into the formulation of packaging. The starch is blended with other bio-based polymers such as guar, konjac glucomannan, carrageenan, chitosan, xanthan gum and gelatin as well as active ingredients such as nanoparticles (NPs), plant extracts and essential oils to develop hybrid biodegradable packaging with reduced water vapor permeability (WVP), low gas transmission, enhanced antimicrobial activity and mechanical properties. Hybrid RS based active packaging is well known for its better film forming properties, crystalline structures, enhanced tensile strength, water resistance and thermal properties. This review concludes that RS, due to its better film forming ability and stability, can be utilized as polymer of choice in the formulation of biodegradable packaging.

## 1 Introduction

Biodegradable packaging is derived from bio-based materials and can be used as an alternative to synthetic plastics due to its ability to degrade naturally and food preservation potential [1]. Among the polysaccharides, starch is amply accessible as one of the economical biodegradable polymers. The conventional sources of starch include potato, rice, cassava, wheat, maize, and corn. There is a need to utilize unconventional starch sources, including low-graded fruits and vegetables as well as their wastes to produce low-cost starch [2,3]. Dumping of such wastes or byproducts raise environmental problems, in addition to loss in the food and agriculture sector [4].

The production of biopolymers has been increased from 300,000 tonnes in 2009 to 2.11 million tonnes in 2019 [5]. Synthetic plastics are impacting biodiversity and implicate substantial socioeconomic costs [6]. Over the years, the production of non-biodegradable plastic is reducing since it is resulting in ecological problems including exhaustion of resources, waste generation and landfills, global warming, and environmental pollution [7]. In 2015, the production of non-biodegradable and biodegradable packaging was 64% and 36%, respectively. By 2019, the fraction of biodegradable packaging reached 56%, and a drop in the production of non-biodegradable plastics was reported [5]. By utilizing circular economy approach, EU commission is targeting 55% of plastic packaging recirculation by 2025, and all plastics will be recyclable or reusable by 2030 [8]. Recently it was reported that biodegradable packaging constitutes a significant (1.14 million tonnes, 53%) proportion of total bioplastic market, especially in food packaging industry [5,9].

Annually, a significant amount of agricultural waste is generated by food processing industries, including seeds, kernels, leaves, stem and roots which are discarded [10,11]. This waste can be one of the potential starch sources. The amylose content in pineapple stem starch (34.4%) is higher compared to the most common starches (16%–30%) [11]. During industrial processing mango kernels are discarded as waste and it is reported to contain 48.43% starch [12]. Jackfruit seeds are comprised mainly of starch with high amylose content ranging from 22.10% to 38.34%. Longan and loquat kernels are potential sources of starch exhibiting around 59% and 71% of starch, respectively [2]. Approximately, 36 million tonnes of banana peels are generated annually containing substantial amounts of starch with high amylose content (25.7%) [4]. In this review, the edible starch sources such as food materials are termed as conventional sources while the starch sources which are non-edible and generally considered as food waste/byproducts are termed as non-conventional sources. The conventional, as well as non-conventional sources of starch with amylose and amylopectin contents are summarized in Table 1.

**Table 1:** Conventional and non-conventional sources of starch with amylose and amylopectin contents.

Starch sources	Conventional sources of starch			
	Starch content (%)	Amylose content (%)	Amylopectin content (%)	References
Potato	60–80	25–31	69–75	[[13], [14], [15]]
Arrowroot	88–89	20.5	79.5	[13,16,17]
Rice	81–92	15–35	65–85	[13,15,18]
Corn	70–75	17–28	75–83	[13,15]
Maize (Normal)	70–72	15–20	80–85	[8,19]
Maize (Hylon 7)	71–87	40–77	23–60	[20,21]
Wheat	60–70	17–34	75–80	[13,15,22]
Cassava	20–32	19–22	28–81	[13,15,23]
Pulses	50–60	36–42	58–64	[8,24]
Yam	16–20	9–15	85–91	[25,15]
Chestnut		15–22	78–85	[8]
	**Non-conventional sources of resistant starch**			
Pineapple stem	77–78	34.4	65.6	[2,11,26]
Mango kernel	65–66	19.2	80.8	[27,28]
Jackfruit seeds	60–80	22–38.3	61.7–78	[2,8,29]
Unripe bananas	65–70	21.5–25.7	74.3–78.5	[4,25]
Loquat seeds	19–20	29–45	51–71	[3,8,30]
Litchi seeds	40–42	16.9	83.1	[31,28]
Avocado seeds	79–81	10–44	56–90	[2,8]

Nowadays, high amylose starch or RS is in great demand as an emerging biopolymer for food packaging due to better mechanical and barrier properties than NS films [32,33]. However, RS based films showed difficulty in film forming process due to the gelatinization of compact starch granules structure as well as poor flexibility due to its strong cohesive energy derived by rich amylose content. One of the proficient strategies reported to increase the film forming characteristics, tensile strength, water resistance and enhancement of crystalline structure, is the integration of RS with other biopolymers such as guar, konjac glucomannan, carrageenan, chitosan, xanthan gum and gelatin [33]. The main objective of this review article is to highlight the types of RS, NS modification methods for the formulation of RS and importance of RS as polymer of choice for biodegradable active food packaging. Previous studies mainly focus on conventional sources of starch however, utilization of unconventional sources of starch such as fruit and vegetable by products can not only pave a path towards food waste reduction but also serve as alternatives for the extraction of low-cost starch, ultimately leading towards the sustainability. In this review nonconventional sources of starch were explored to emphasize the extraction of starch from low-cost raw materials.

## 2 Methodology

**Fig. 1:**
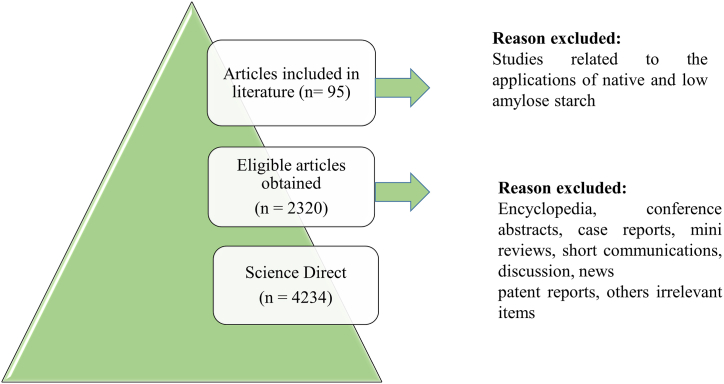
PRISMA diagram for literature review.

Published literature in ScienceDirect database (https://www.sciencedirect.com/) on resistant starch as a polymer of choice for biodegradable active food packaging was referred. The studies were consulted from 2012 to 2022. Key words “high amylose starch”, “resistant starch” and “modified starch” were applied to carryout advanced search string of the database to evaluate research documents (research papers, review papers and book chapters), having these search terms in title, abstract or keywords. Similarly, to highlight the methodologies used to produce high amylose starch in past years, key words such as “chemical modification”, “enzymatic modification”, “physical modification” and “genetic modification” were utilized. Moreover, key word “biodegradable active food packaging” was considered to determine the effect of active biodegradable packaging on the quality of food commodities over the certain period of time. Preferred Reporting Item for Systematic Reviews and Meta-Analysis (PRISMA) method (Fig. 1) was used by selecting ScienceDirect database. Based on the objectives, year of publication and research results, the selected articles were reviewed and summarized. The inclusion criteria included: a) research on high amylose starch b) modification methods and types of modified starch, c) its application to produce active food packaging d), published in the form of research article, review article and book chapters. The exclusion criteria involved: a) research on native and low amylose starches, b) conference abstracts, and short communication. The search process commenced by reviewing the titles and abstracts of search results and evaluating those on the set criteria. The database search with all keywords yielded 4234 search results, out of which 2320 articles (research article, review article and book chapters) were obtained. Based on the selected criteria, only 95 research documents (articles, review articles and book chapters) were selected to describe the types of RS, modification methods for the development of RS and its importance as the polymer of choice for biodegradable active food packaging.

## 3 Composition of resistant starch

Among the polysaccharides, starch is an important natural polymer which exhibits the properties to be utilized as the food packaging ingredient. Based on the composition, starch contains around 15–25% of linear amylose (poly-α-1,4-d-glucopyranoside) and the rest is composed of highly branched structured amylopectin (poly-α-1,4-d-glucopyranoside and α-1,6-d-glucopyranoside) along with minute amount of proteins and lipids [9,25,34]. Starch in its natural state demonstrates limited properties including poor mechanical properties (tensile strength, elasticity modulus, shear stress and strain at break), high water vapor permeability due to its hydrophilic nature, and poor processability to form starch-based packaging films [35]. To enhance properties of NS and to meet the technological demands, the researchers were eager to develop modified starch with advanced properties [36]. This was achieved by using various modification methods broadly categorized as; chemical, enzymatic, physical and genetic modifications [37].

RS is non-digestible starch due to its high amylose content and acts as a dietary fiber [38]. The amount of amylose is dependent on various variables including, availability of starch content in a food commodity, amylose-amylopectin ratio, granule type and starch crystallinity along with modification method utilized to obtain high amylose starch [39].

## 4 Types of resistant starch

Depending on physical and chemical properties, RS is classified into five types: RS1, RS2, RS3, RS4 and RS5, respectively [40,41]. RS1 also known as encapsulated starch is entrapped within plant cells or polymer matrix. It is reported that slow rate of starch hydrolysis involves three major mechanisms namely, restricting enzymatic hydrolysis, lessening starch damage, and improving thermal resistance respectively [40]. RS1 is mostly obtained from whole grains, coarsely milled kernels, pulses/legumes, or intact plant cells [41]. RS2 is in the form of raw intact starch granules which cannot be gelatinized and accessed by digestive enzymes because of its compact structure, therefore it is called resistant granules. Green bananas, some legumes, and raw potatoes are the rich sources for RS2. RS3 mostly results from the moist-heat food processing and is called retrograded starch. After gelatinization, the retrogradation process results in recrystallization of few single chains to double helices via hydrogen bonds [41]. Cooked and cooled potatoes, bread, cornﬂakes, cooled yam, cassava and food products with repeated moist heat treatment are the sources of RS3 [42]. Chemical modifications of NS such as cross linking, etherification and oxidation produce type 4 RS or chemically modified starch [41,43]. Processed food products such as cakes and breads are the source of RS4 [42]. RS5 is a type of RS which is formed due to amylose-lipid complexes. By reducing the swelling of granules during cooking, the resistance for hydrolytic enzymes increases due to the presence of these amylose lipid complex in starch granules. Generally, these complexes are formed during food processing; however, they can also be prepared under controlled conditions [40]. The formation of amylose-lipid complexes is inﬂuenced by the amylose-amylopectin ratio of starch and botanical source [41]. The characteristics and sources of different types of RS are summarized in Table 2.

**Table 2:** Characteristics and sources of different types of resistant starch [[40], [41], [42]].

Types of resistant starch	Description	Sources	Characteristics
RS1	Encapsulated starch	Whole grains, coarsely milled kernels, pulses/legumes, or intact plant cells	Slow rate of starch hydrolysis, restrict enzymatic hydrolysis, lessening starch damage, improving thermal resistance
RS2	Intact granular starch	Green bananas, some legumes, high amylose starch and raw potatoes	Cannot be gelatinized and inaccessible to digestive enzymes
RS3	Retrograded amylose	Cooked and cooled potatoes, bread, cornﬂakes, cooled yam, cassava and food products with repeated moist heat treatment	Results from moist-heat food processing, the retrogradation process results in recrystallization of few single chains to double helices via hydrogen bonds
RS4	Chemically modified starch	Not naturally occurring; processed food products such as cakes and breads	Cross linking, etherification and oxidation of native starch
RS5	Amylose-lipid complexes	High amylose starches and botanical sources	Amylose lipid complex in increases resistance to enzymes by reducing the swelling of granules during cooking, potential health benefits in glycemic response as well as colon cancer.

## 5 Properties of resistant starch

Various health benefits are associated with RS due to its relative resistance towards enzymatic and temperature treatments. Due to the high amylose content, RS exhibits hydrophobic properties and low water-binding properties [43]. In addition to this, high-amylose starch demonstrates several physicochemical characteristics, including ease of retrogradation, excellent gelling strength, increased swelling, and viscosity, as well as high film-forming ability. These predominant characteristics of RS extend its industrial applications, such as the development of adhesives, papers and as an excellent alternative polymer of choice to produce biodegradable food packaging [44].

**Fig. 2:**
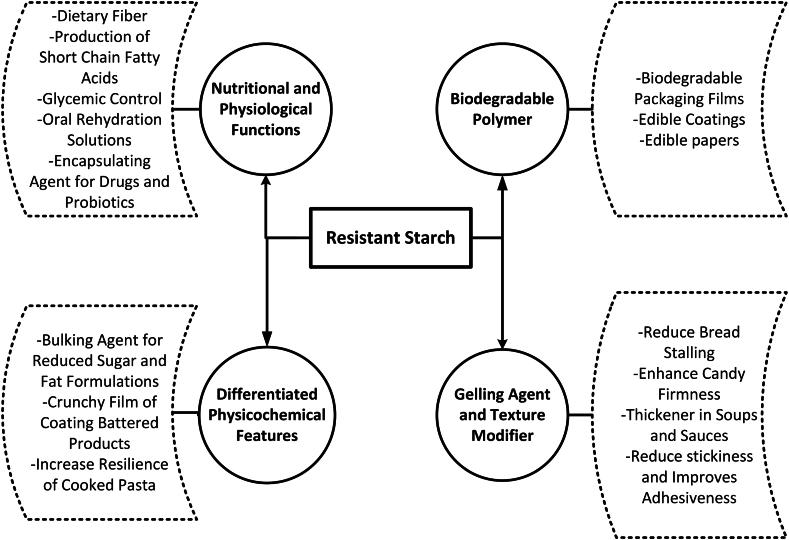
Potential industrial applications of resistant starch.

Apart from its industrial applications, high amylose starches are known to impart health benefits on the consumers as a potential prebiotic [45]. RS is a functional food ingredient and can be used to replace the conventional flours to develop fiber rich food products with improved nutritional and low caloric value [42]. It exerts positive effects on the gut microflora, digestive functioning as well as control the diabetes and cholesterol of the consumers. RS have low water binding capacity and can be fused into food products up to the concentration of 30% to control the food processing conditions, such as moisture content, water solubility, and sensory characteristics [41]. In comparison to conventional high-fiber products, RS can produce low-bulk high-fiber products with improved appearance and texture [39]. The potential applications of RS are depicted in Fig. 2.

## 6 Modification methods to produce high amylose starch

**Fig. 3:**
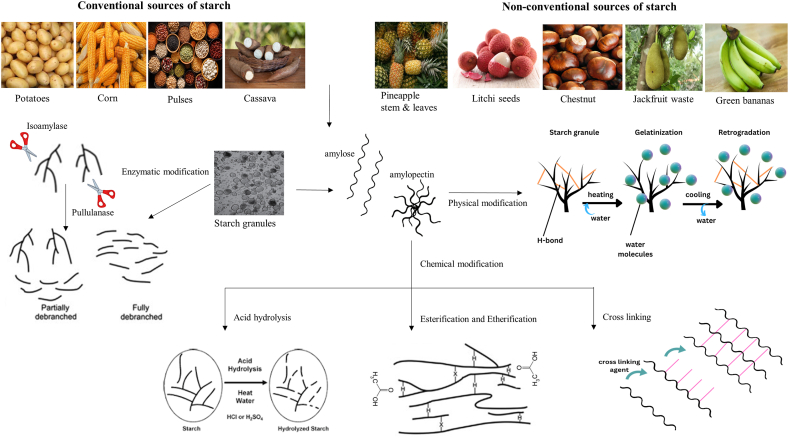
Methods for the modification of native starch into resistant starch.

The NS modification methods to produce high amylose starch are usually characterized into four main categories including chemical, enzymatic, physical and genetic modifications (Fig. 3) [3,41].

### 6.1 Chemical modification

Chemical modification targets the addition or substitution of functional groups of NS to improvise its molecular structure to prevent enzymatic digestion. Under the category of chemical modification, mainly three processes are utilized to produce RS namely cross linking, esterification and etherification, and acid treatment [46,47].

#### 6.1.1 Cross linking

Cross-linking is a type of chemical modification which is carried out by treating the NS with various reagents to integrate ether or ester linkage by substituting hydroxyl group of starch with phosphate, acetyl, hydroxypropyl, octenylsuccinyl and citryl groups respectively [41,48]. One of the predominant and earliest methods for the modification of NS is phosphorylation which is of great interest for food industry. The reaction produces cross linked derivative namely monostarch phosphate or distarch phosphate based on reaction conditions [49]. Phosphate replacement on amylose or outer branches of amylopectin produces steric interference causing hindrance in linearity of molecular chains [41]. Phosphate modification displays improved characteristics such as thermal stability, resistance to low pH, high shear, improved textural and viscosity [50].

Reaction with succinic anhydride and octenylsuccinic anhydride (OSA) is another way to chemically modify NS. During the reaction, the anhydride ring of OSA breakdown and produces an ester with a starch hydroxyl group. Cross linked starch is formed from the left-over carboxylic acid group from anhydride through esterification process [47]. Modification of starch with an ionic substituent group such as succinate weakens the internal bonding which clasps the granules together. Along with this starch succinate presents anticipated features including reduced tendency to retrograde, low-temperature stability, and low gelatinization temperature, high-thickening power, good film-forming and biodegradable properties [50].

Cross linking improves the resistance of starch as well as strengthen and stabilize the NS derivatives by randomly adding inter- and intramolecular bonds [47]. The functional and chemical properties of cross-linked starch are influenced by various reaction parameters including temperature, pH, time, source of starch, concentration and type of cross-linking reagent. By using double modification i. e cross linking and esterification, there was an increase in the RS content from 21.49% to 29.14% in banana starch [41].

#### 6.1.2 Esterification and etherification

By using this method, hydrophilic groups of NS are replaced with hydrophobic groups leading to hyproxypropylation and acetylation [47]. In acetylation, hydrophilic hydroxyl groups are replaced with hydrophobic acetyl groups by using acetic anhydride and vinyl acetate. Acetylation halts hydrogen bonding between hydroxyl groups and water molecules [50]. Along with this, acetylation hinders or reduces crystallization or retrogradation which influence the stabilization of the starch solution. Substitution with acetyl group prevents association of amylopectin outer branches [37]. The acetyl groups generate steric forces due to which digestive enzymes are incompetent to appropriately bind to the starch [47]. Acetylation of rice starch improved the characteristics such as higher elongation, increase resistance to thermal degradation and water solubility [51].

By etherification of NS, hydroxypropylated starches are usually formed with propylene oxide in the presence of an alkaline catalyst [41]. The hydroxypropyl groups cause disruption in the inter- and intra-molecular hydrogen bonds of NS chains. This deteriorates the starch granular structure which confers the resistance to starch digestion [37].

#### 6.1.3 Acid treatment

The target of acid treatment is to hydrolyze the amorphous parts of starch granules followed by hydrolysis of crystalline region [46]. Acid modiﬁcation followed by autoclaving and retrogradation upturn the RS content [41]. The short chains of amylopectin realign to generate highly ordered double helix structures during retrogradation which can resist amylolytic digestion [49]. Acids such as sulfuric acid, hydrochloric acid, and orthophosphoric acid are primarily utilized for the alteration in the molecular structure as well as physiochemical properties of NS [37].

Although chemical modifications have been a successful technique to enhance the RS content, the extensive use of chemical reagents and chemically modified ingredients in food needs to be regulated within safe consumption amount [52]. Along with this, chemical modification of starch is associated with long reaction times and poses harmful effects on the environment if the use of excess chemicals would not be dealt cautiously [53].

### 6.2 Enzymatic modification

Enzymatic modification of NS involves the debranching of amylopectin with enzymes such as isoamylase and pullulanase which increase the RS content [50]. Enzyme treatment followed by retrogradation results in the formation of more compactly bound crystalline structures of amylose. Due to the change in molecular structure and physiochemical properties, amylose becomes resistant to enzymatic digestion [41]. Both isoamylase and pullulanase solely act on the branch chains with α-(1,6)-glycosidic bonds present on amylopectin which results in the improvement of amylose content [41]. Mostly high content of type III RS crystals is produced by debranching of amylopectin followed by retrogradation [46]. Multiple dynamics including temperature, pH and choice of enzyme play critical role in enzymatic modification of NS.

### 6.3 Physical modifications

Physical modification is classified into thermal modification, and non-thermal modifications. Hydrothermal treatment implicates physical modiﬁcations that cause a change in physico-chemical properties of starch without modifying its granular structure [54]. Hydrothermal treatment is categorized into two primary streams namely annealing and heat moisture treatment [41]. Hydrothermal treatment targets to improve the degree of starch crystallinity, arrange starch chains in crystalline and amorphous layers as well as fortify crystalline form of granules. These physical modifications change the chemical properties such as reduced swelling ability and solubility in turn, increasing the resistance of starch granules to amylolytic enzyme [40,42].

Autoclave technology, followed by the retrogradation, proved to increase the RS content. In this method, starch is gelatinized at temperatures above 100 °C under pressure, which causes the complete disruption of starch granules. Upon cooling, the amylose chains form hydrogen bond to produce stabilized double helices (RS3 crystallites). In general, the amount of RS is proportional to number of autoclave/retrogradation cycles [41].

The amount of RS can also be enhanced by using a combination of starch gelatinization followed by enzymatic debranching of gelatinized polymer. Later, the debranching enzyme is deactivated, the product is isolated and obtained by using drying/extrusion/cocrystallization processes. RS produced by using heating-cooling cycles is RS3. Starch solution autoclaving (30% w/v) at 121 °C for 1 h and cooling at room temperature followed by storage at 48 °C for 1 day produce high RS3 [54].

Extrusion enhances the RS levels by using high shear mechanisms which cause depolymerization of the starch granules. This results in the straightening of chains that are more susceptible to retrograde into RS3 [47]. Acid hydrolysis followed by low shear extrusion improved the RS content of corn NS from 11% to 20% [41,54].

### 6.4 Genetic modification

Chemical and physical modifications play a vital role in achieving the desired characteristics of high amylose starch however, these procedures limit the acceptability by the consumer due to lack of clean labelling. Therefore, currently biological methods are of great interest to enhance the starch amylose amount. Genetic modification can be carried out by using interbreeding of high amylose varieties and suppressing the expression of starch-branching [40,31].

#### 6.4.1 Interbreeding of high amylose varieties

During this type of genetic manipulation, mutants containing genes for high amylose production are interbred. Native corn contained a recessive *amylose extender* (*ae*) gene which was interbred over time to produce high amylose corn starch types such as Hylon 5 and Hylon 7. The *ae* gene mutants are reported to produce 85.6% amylose [47]. Likewise, by inbreeding recessive genes in barely, rice and peas also reported to have enhanced levels of amylose yield [55].

#### 6.4.2 Inhibition of starch-branching enzymes

Granule bound starch synthase I (GBSSI) is involved in the synthesis of amylose by extending α-1,4 linkages of glucose polymers. In case of amylopectin production, three enzymes are involved; starch synthases (SSs), starch branching enzymes (SBEs), and debranching enzymes (DBEs) [34].

Amylose content can be enhanced in cereals by boosting the GBSSI expression since this enzyme is directly involved in the synthesis of amylose [56]. However, this methodology has primarily two limiting factors, deficient non reducing ends in amylose and substrate competition between amylose and amylopectin [47]. Amylose content is not always significant with respect to the activity of GBBS since it has limiting activity in the starch granule [57]. Therefore, during this procedure amylose content no longer proliferates in GBSSI-enhanced lines, once an optimum level is attained. As an alternative to this is the inhibition of enzymes such as starch synthases II a (SSIIa), SBEs and other enzymes taking part in the synthesis of amylopectin [58]. Suppressing SBEs, especially the SBEIIb homologue, produces an α-glucan with only a few branches attached to partial backbone chains, providing efficient substrates for the SSIII homologue for successive elongation [36]. In order to edit or knock out, SBEI and SBRII gene CRISPR/Cas technique is utilized targeting full allelic function loss. This genetic engineering technique appeared to produce crop lines with highest amylose content [36,59].

### 6.5 Dual starch modification methods

To obtain high amylose starches and modulate the physiochemical properties of NS, dual modification methods offer a promising pathway. Generally, in dual modification of starch, the first modification is used for the preparation of granular surfaces in the starch. Subsequently, the second modification targets the bond strengthening in the starch chains. Dual modification is broadly classified into two categories; homogenous dual modification and heterogenous dual modification. Dual physical, enzymatic and chemical alterations fall under homogenous modifications. Dual chemical/physical, chemical/enzymatic or the reversed dual modification processes are the types of heterogenous modifications [60].

The combined effect of extrusion and heat moisture treatment of corn starch produced high amylose starch with increased gelatinization, thermal stability, crystallinity, and resistance to enzyme digestibility [61]. The synergistic effect of dry heating and annealing caused structural and functional reorganization of starch, in return altering its molar mass and amylose-amylopectin ratio, which resulted in high pasting temperature and stability of starch. Moreover, the dual modification reduced the enzymatic digestibility due to elevated levels of RS [62]. The combination of annealing and ultrasonication lead to alteration of inter and intra hydrogen bonds as well as reassociation of amylose and amylopectin chains in millet starch. This resulted in enhanced RS levels up to 31–33% along with reduced water absorption capacity [63].

## 7 Importance of resistant starch in food packaging

Due to increasing environmental hazards and concerns for human health associated with synthetic food packaging, extensive research is being carried out on biodegradable food packaging and its applications in food industry. NS is used in packaging because of its vast availability, low cost and film forming capacity. However, the application of NS is hindered in food packaging industry because of its high moisture sensitivity and hydrophilic nature [41]. To encounter this problem, RS based biodegradable packaging is getting attention by incorporating other biopolymers as well as active ingredients such as NPs, plant extracts and essential oils [64]. RS is recognized for its hydrophobic nature and low water binding properties due to high amylose content [43]. This makes hybrid RS food packaging promising in enhancing the shelf life of food commodities due to its capabilities to decrease water vapor permeability (WVP), gas transmission, as well as to enhance the antimicrobial and antioxidant properties [58]. Strong internal associations because of hydrogen bonds between the chains of RS are responsible for limited water uptake and high temperature required for complete galvanization [65]. Hybrid RS based packaging is known due to its better film forming properties, enhanced chemical and crystalline structures, tensile strength, water resistance and thermal properties (Table 3) [33].

**Table 3:** Characteristics of resistant starch based composite biodegradable packaging films.

Starch source	Blended polymer/active ingredient	Film preparation technique	Inference	References
High amylose corn starch	Gelatin	Casting	Significant increase in film mechanical strength. Increased the thickness and transparency of the prepared films. Decreased film solubility which improved its water resistance and water vapor permeability. Improved thermal stability of the composited films.	[66]
High amylose cornstarch with amylose-amylopectin ratio of 80:20	None blended polymer or active ingredient was incorporated rather plasticizers glycerol and xylitol were added only.	Casting	The HA-glycerol films retained the highest moisture content. The HA films exhibited higher glass transition temperature, higher tensile strength, higher modulus of elasticity and lower elongation at break. The tensile strength and modulus of elasticity decreased and the elongation increased with increasing plasticizer concentrations above 15% on dry solid basis.	[67]
High amylose cornstarch with amylose-amylopectin ratio of 80:20	Chitosan	Casting	Significant increase in film mechanical strength. Increased the thickness and transparency of the prepared films. Decreased film solubility which improved its water resistance and water vapor permeability. Improved thermal stability of the composited films	[68]
High amylose corn starch	No other polymer or active ingredient added. Plasticizer: glycerol	Extrusion along with casting	It was observed that when the glycerol content decreased, the highest values of the puncture strength (15.02 ± 3.14 N). Increment in deformation of edible films when glycerol content was increased. Water permeability vapor of the edible films increased in proportion to the glycerol content. When the extrusion temperature increased and glycerol content decreased, the water solubility increases significantly. Edible films prepared from an extruded formulation of high amylose corn starch and glycerol was an effective treatment to maintain physical and chemical quality of mango at 12 °C for 16 days.	[69]
Mung bean starch	PVA/malic acid and succinic acid	Casting	MBS films cross-linked by SA showed lower swelling power and WVP values than those by MA. The addition of MA and SA resulted in the decrease of degree of crystallinity. Addition of MA and SA into MBS led to smoother structure compared to the native MBS film. Higher acidity in MA resulting in more hydrolyzed MBS molecules, led to lower tensile strength than that in SA Mechanical properties of native and cross-linked MBS film were found to decrease after soil burial test, confirming its biodegradability	[70]
High amylose corn starch	Konjac glucomannan	Casting	Synergistic effect between HCS and KGM enhanced film-forming properties of high amylose starch. Physicochemical properties of the films including X-ray diffraction, Fourier transform infrared spectroscopy, scanning electron microscopy were improved. Exhibited higher glass transition temperature. Increase water resistance and water vapor permeability. The crystallinity and the proportion of short-range order structure of the films increased. The micromorphology of the films exhibited more even texture after KGM was incorporated in. The tensile strength, elongation at break were also improved significantly.	[33]
High-amylose starch	PVA and nano-TiO_2_	Casting	Significant increase in film mechanical strength. Increased the thickness, higher modulus of elasticity and lowered elongation at break. Transparency of the prepared films increased. Decreased film solubility which improved its water resistance and water vapor permeability. Improved thermal stability of the composited films. The crystallinity and structure of the films increased. Exhibited antimicrobial activity.	[32]

## 8 Properties of resistant starch based food packaging

### 8.1 Thickness and mechanical properties

The thickness and mechanical properties of hybrid RS based biodegradable packaging is enhanced due to the influence of high amylose content. Generally, an increase in amylose content can provide a higher amount of hydroxyl groups for hydrogen bonding leading to entangled chain networks and double helical junctions which can cause the film thickness and tensile strength to increase while elongation at break can decrease. The tensile strength of packaging film increases as the amylose content increases, however up to a limited point. As the critical limit of amylose content exceeds, the problem of brittleness is encountered due to amorphous regions of amylose chains. To enhance the plasticity of films, plasticizers such as glycerol and sorbitol are added [71,72]. The thickness of films is also dependent on the solid content of film forming solution [73]. RS based films are incorporated with other biopolymers to enhance the mechanical, barrier and preservation potential, such as guar, konjac glucomannan, carrageenan, chitosan, xanthan gum and gelatin [33]as well as active ingredients including NPs, plant extracts and essential oils [64,74]. The films blended with konjac glucomannan enhanced the thickness from 0.104 mm to 0.114 mm indicating that polysaccharide decreased the bulk density of starch film due to the co-association of konjac glucomannan with starch chains [33]. Hybrid RS/chitosan-based films [68] and high amylose corn starch/gelatin-based films (Gel-Sha) [58] showed significant increase in film mechanical strength and thickness due to the volume expansion and swelling of the starch component.

The mechanical properties of RS-based films are dependent on various factors including crystalline structure, plasticizer type, amylose content, and the extent of plasticization of the amorphous parts [75]. The tensile strength of high amylose starch packaging films plasticized by glycerol was higher than the high amylose starch films plasticized by xylitol. Furthermore, glycerol and xylitol can exert an anti-plasticizing effect on the tensile strength of the starch (both NS and RS) films beyond the critical limit [67].

**Fig. 4:**
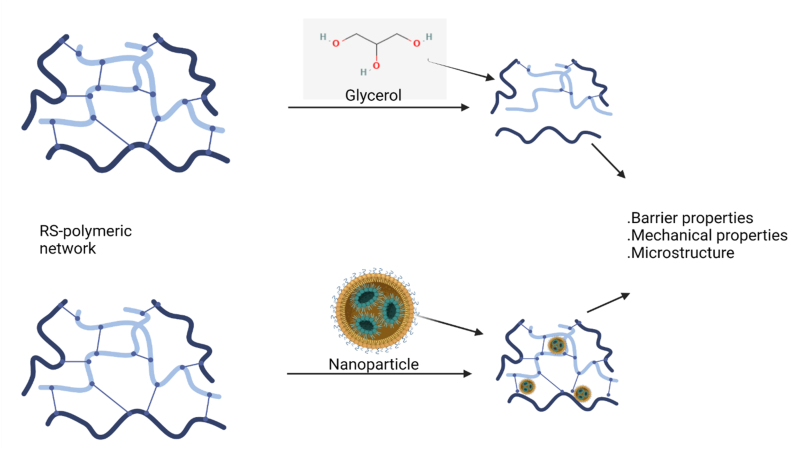
Effect of glycerol (plasticizer) and nanoparticles on the structure and properties of RS-based films (created with Biorender.com and PubChem).

Starch-starch interactions necessary to absorb mechanical energy are restricted at low concentrations of plasticizer as its molecules are strongly attached to the starch. This phenomenon was similarly observed by Ref. [76], who observed that plasticizers with low molecular mass (i.e., glycerol) can easily create intermolecular spaces between starch polymeric networks and can reduce the number of hydrogen bonds attached to the starch chains (Fig. 4) leading to reduced puncture strength as compared to high molecular mass plasticizers (i.e., sorbitol and xylitol).

Hybrid RS based films further enhance the mechanical properties of films. Generally, at initial concentrations, hybrid biopolymers and NPs improve the mechanical characteristics as high amylose starch molecules are separated and reduce the cohesion forces between starch chains [33]. As the optimum range exceeds, the mechanical properties begin to decrease. Liu et al. [32] explained when nano-TiO_2_ content in the film was increased from 0 to 0.6%, the tensile strength of the film remarkably increased from 3.66 MPa to 9.53 MPa. The formation of hydrogen and covalent bonds with nano-TiO_2_ and high-amylose starch/PVA enhanced the molecular chain rigidity of high-amylose starch/PVA. When the nano-TiO_2_ content exceeded 0.6%, the tensile strength of the blend films showed a slight decline due to the agglomeration and jamming of nano-TiO_2_. The elongation at break of the blend films decreased slowly from 62.74 to 31.71% along with an increase of nano-TiO_2_ content [32].

### 8.2 Barrier properties

Due to high amylose content, RS is recognized for its hydrophobic nature and low water-binding properties [41]. Generally, strong internal associations between the chains of RS due to hydrogen bonding are responsible for limited water uptake [65]. Since WVP occurs from the hydrophilic part of the film and thus is dependent upon the hydrophilic/hydrophobic constitutes of the starch films. RS based films exhibit high oxygen barrier properties to reduce respiration and delay oxidation which is of great importance in maintaining shelf life of food commodities. Moreover, the barrier properties of RS films are based upon the parameters such as nature, concentration and properties of other biopolymers/active ingredients added, amount of plasticizer, film thickness, and water activity [75]. For instance, three-dimensional organizational network of RS film is changed after the addition of plasticizers into the matrix causing an increase in free volume of the system by reducing the intermolecular attractive forces, consequently, the network becomes less dense which causes the permeation of water molecules and gas vapors [77]. On the other hand, the addition of active fillers (NPs or bioactives) in the starch matrix can increase the effective path length by creating a tortuous pathway for the diffusion of water and gas molecules [74]. Furthermore, the mass transfer of gases in a semi-crystalline polymer like RS is influenced by the polymer nature [78].

The modified starch prepared by extrusion process was used to formulate high amylose corn starch based edible films [69]. The results showed that the WVP of the edible film increased in proportion to the glycerol content. The hydrogen bonds, ionic and van der Waals forces between biopolymers reduce by the addition of glycerol, hence, increasing the free space between the chains. This increases water diffusion into the film matrix therefore, there is an increase in the WVP of plasticized films. By adding 0–30% (w/w) glycerol or 0–60% (w/w) sorbitol into mung bean starch (MBS), the hydrophilic nature of both plasticizers favored the adsorption of water molecules, thus increasing the water vapor permeability [79]. Incorporation of malic acid (MA) and succinic acid (SA) with concentration from 10 to 30%, WVP values slightly decreased. Formation of hydrophobic ester bonds and covalent network generated more hydrophobicity via cross-linking that reduced the capacity of water vapor transmission [70].

WVP results are also related to the degree of swelling power. MBS is hydrophilic in nature due to the presence of free hydroxyl groups on d-glucopyranose ring. These free hydroxyl groups of MBS and carboxyl group of cross linkers (MA and SA) are involved in esterification. As the ester bond increases, free hydroxyl groups of the MBS molecule decrease. MBS films incorporated by MA exhibited a greater degree of swelling power than that of SA, at the same acid content. The results suggested that MBS film cross-linked with MA created lower ester linkage due to the lower pKa value of MA, resulting in more hydrolysis of the glycosidic linkages in MBS and leading to more hydrophilic nature. In addition, a higher degree of swelling power of MBS film with MA could also arise from steric hindrance by the presence of hydroxyl group in MA, probably leading to a lower degree of cross-linking [70].

### 8.3 Crystalline and chemical structure

X-ray diffraction (XRD) analysis is carried out to study the amorphous and crystalline structure of the biopolymers used as film materials [73]. Native starch is semi-crystalline and according to the sources, displays different crystalline patterns (A, B, C, and V). The double-packed helix indicates A, B, and C-type crystals [80]. For instance, the presence of peaks between 11 and 24° indicates type A, B, and C starches. On the other hand, a single helical structure promotes V-type conformation which results from the complex formation of amylose with certain film formulation components [20]. The crystallinity of the film increases primarily after the incorporation of other biopolymers or NPs [32]. However, beyond the critical limit, the crystallinity of the composite film can decrease which may be associated to the interaction between amylose and hybrid polymers/active ingredients [33]. Similarly, the addition of plasticizers can also influence the crystallinity of RS by reducing the intensity of the peaks. For instance, glycerol can block the rearrangement of starch segments by forming hydrogen bonds with the hydroxyl groups on starch chains thus preventing the formation of crystals on nucleus [20]. Zou et al. [33] reported that the high amylose corn starch granule displayed typical B-type crystalline structure with diffraction peaks at 5.6°, 17°, 22° and 24° and V-type crystalline structure with a peak at 19.8°. The diffraction peak at 24° disappeared in the high amylose corn starch film, which reflected the granular structure. The crystallinity of potato starch/cellulose nano fibrils-based films was increased from 42.00 % to 75.38% due to strong intermolecular hydrogen bonding and van der Waals force of attraction in cellulose [81]. Starch nanocrystals of rice and potato enhanced the crystallinity more than three times as compared to native rice and potato starch [82]. Native starch was modified by undergoing acid hydrolysis to obtain starch nanocrystals. Therefore, the increase in crystallinity of starch nanoparticles is associated to the hydrolytic action of the acid that breaks the amorphous regions of lower-density starches. However, the modification of native starch did not alter the type of crystallinity which remained similar to type A and type B characteristics [82].

Scanning electron microscopy (SEM) is of great importance to determine the microstructural changes in films and to evaluate cross-sectional and surface topography [73]. Generally, RS based films are uniform, smooth, and homogenous without any cracks or pores even after modification which is an indication that the gelatinization step was sufficient to disrupt all starch granules. The addition of NPs up to a certain extent also doesn't have any significant influence of the microstructure of RS films, however, at higher concentrations it can lead to agglomerate formation making the resultant films less cohesive and more porous [82,83]. Liu et al. [32] stated that the high-amylose starch/PVA blend films with 0.8% TiO_2_ NPs were smoother and more compact. TiO_2_ NPs play the role of the crosslinking agent, which functionally enhance the affinity of high-amylose starch and PVA by making the film surface smoother and denser as compared to the control film. Martins et al. [82] explored the impact of rice and potato starch nanocrystals on the functional properties of native and hydrolyzed rice starch-based films [82]. The addition of 0.3% rice starch nanocrystals and 0.1% potato starch nanocrystals to native and hydrolyzed rice starch-based films gave a fine, smooth and cohesive film surface in comparison to the rough and fractured surface of control films [82].

It is also important to investigate the chemical fingerprinting of the biopolymeric films as it can be useful in understanding the chemical interactions between the film components and identifying the functional groups of interest. Several characteristics peaks can be observed in the FTIR spectra of RS based films at 3300 cm^−1^ and 1600 cm^−1^ (O–H stretching vibrations), 2900 cm^−1^ (C–H stretching vibrations), 1100 cm^−1^ (C–O stretching), 1080-1040 cm^−1^ (C–*O*–C group stretching in the anhydro glucose unit) [[84], [85], [86]]. However, after the addition of plasticizers or other additives the characteristic peaks can shift to higher or lower wavenumber depending upon the type of interaction between the starch chains and additives. For instance, the addition of glycerin in potato starch films shifted the characteristic bands from 3450 cm^−1^ and 1170 cm^−1^ to a lower wavenumber while 1040 cm^−1^ to a higher wavenumber due to the formation of hydrogen bonds. Similarly, the addition of TiO_2_ NPs in the starch films led to the formation of new hydrogen bonds which moved the O–H spectra from 3300 cm^−1^ to 3267 cm^−1^ [86].

### 8.4 Thermal properties

The thermal property of film is influenced by the polymer's rubbery or glassy state. The glass transition temperature (Tg) of the packaging films is an important parameter for the selection of processing and storage conditions of the film along with its application. Differential scanning calorimetry (DSC) is generally used to validate the Tg of the materials [73]. Mostly, Tg of the RS films ranged between 55 and 80 °C and is influenced by RS source, plasticizer nature and its concentration, and additive type [86]. The increase in Tg due to the addition of various NPs can be due to strong interactions of nanocrystals with the polymeric chains forming a 3D network with improved thermal resistance [68]. For instance, an improvement in the Tg of the native starch films (from 55 to 72 °C) was observed with the addition of starch nanocrystals (from 0.1 to 0.3%) due to strong interaction between nanocrystals and film matrix [82]. On the other hand, plasticizers like glycerol can reduce the overall crystallinity of the matrix and thus can reduce the Tg of the RS films [82].

### 8.5 Antibacterial activity

Biologically, RS based films do not exhibit antibacterial activity and are generally regarded as non-toxic. However, the addition of active substances such as NPs, plant extracts, and essential oils potentiate antibacterial characteristics in hybrid RS food packaging. RS-based matrices control the release behavior and diffusion rate of the active substances [75]. The nano-TiO_2_ films showed relatively fair antibacterial activity against Gram-positive (*S. aureus*) and Gram-negative (*E. coli*) bacteria. Increasing the nano TiO_2_ concentration, increased the inhibition zone of the films against targeted microorganisms. TiO_2_ can generate strong oxidizing power. Reactive oxygen species (ROS) are generated which interact with the cell wall and cell membrane of the bacteria as well as to its enzymes, DNA, and other lipid and protein content resulting in bacterial cell death. Hence, TiO_2_ can restrict the growth of bacteria. Nano-TiO_2_ exhibit bacteriostatic ability because the inhibition zone will gradually disappear as incubation time go by Ref. [32]. Hydroxypropyl RS based films prepared with the incorporation of pomegranate peel powder acted as antibacterial agent against both *S*. *aureus* and *Salmonella* spp. The inhibition zone against both bacteria increased by increasing the amount of pomegranate peel powder. The pomegranate peel has phenolic compounds which exhibit antibacterial activity and release slowly because of interaction between the hydroxyl groups of phenolic compounds and RS [87,88].

### 8.6 Biodegradation of resistant starch composite films

Biodegradation of the biopolymeric composite films is influenced by the polymer type, additive nature, and the state of the polymer upon film formulation [89]. In literature, the biodegradability of RS films was assessed by various methods i.e., soil burial, microbial activity, and anaerobic test [90]. The increased degree of acetylation for starch films promoted a greater degree of biodegradability by an increase in CO_2_ rate. The insertion of acetyl groups in the polymeric chains might reduce the intra or intermolecular interactions resulting in the increased moisture content. This in turn elevated the rate of microorganism attack on the RS film surface during the soil burial [70,90]. The test was carried out over a period of 5 and 10 days in the laboratory. Mechanical properties of native and cross-linked MBS film decreased after soil burial test. This could be due to the adsorption of moisture from soil, so the degradation occurred by hydrolysis and microorganisms. MBS films cross-linked by MA and SA could also biodegrade, confirmed by the decreased of tensile properties [70]. Plasticized starch was reported to biodegrade completely in 40–50 days, however, the incorporation of chitosan NPs acted as the reinforcing agent, leading to an increase in the stability of composite films which delayed the biodegradation (50–60 days) [91].

## 9 Resistant starch based intelligent packaging

Unlike active packaging, intelligent packaging also known as smart packaging that monitor the quality and safety of a food commodity with reference to its real time environment [92]. The parameter of smart packaging includes freshness, pH and time-temperature indicators. Although RS is one of the potential polymeric candidates, yet there is limited literature available on the preparation of RS based intelligent packaging, its characterization and application [93]. Potato starch based colorimetric pH indicator films were developed and the films produced a color shift from red to green upon the deterioration of pork [94]. A similar study was conducted in which hybrid corn starch/chitosan/purple cabbage extract was developed in order to monitor the fish decay [95]. Yam starch/xanthan gum based intelligent packaging was developed by incorporating red cabbage anthocyanin and beetroot. Under acidic conditions, red cabbage-based hybrid film turned from purple to deep pink while beetroot-based hybrid packaging produced a color change from red to brown. In basic conditions, the hybrid films incorporated with red cabbage and beet root extracts gave a color shift from purple to green and red to yellow, respectively [96]. RS from different sources has a potential to be used in the development of intelligent packaging due to its better film forming ability and stability.

In this study ScienceDirect was only used as a data source to extract the information, however future studies may include data from different sources for comprehensive analysis of RS based literature.

## 10 Conclusion

Food and agriculture byproducts are the potential sources of RS which can serve as a packaging polymer to overcome existing challenges that are associated with high cost of biodegradable polymers. Various modification methods have been developed to produce high amylose starch which can be used to formulate biodegradable films with improved characteristics. NS based packaging lacks stability, has poor barrier properties and thermal stability. However, RS-based film exhibits excellent barrier properties and heat-sealing capacity. RS based active and smart packaging can be developed by incorporating various additives and active ingredients in RS film forming matrix due to high compatibility of RS. Due to high stability and mechanical properties, RS can be a polymer of choice for the development of intelligent packaging. The utilization of low cost and sustainable sources of starch and dual starch modification methods can ensure the availability of stable and economical biopolymer in the formulation of RS based biodegradable packaging which can serve as an alternate to synthetic plastics. Due to its edible nature and compatibility with other ingredients, RS can be used for the packaging of food and pharmaceutical products.

## Data availability statement

Data included in article/supp. material/referenced in article.

## Funding statement

This research did not receive any specific grant from funding agencies in the public, commercial, or not-for-profit sectors.

## CRediT authorship contribution statement

**Saeeda Fatima:** Writing – original draft, Methodology, Formal analysis, Data curation. **Muhammad Rehan Khan:** Data curation, Conceptualization. **Imran Ahmad:** Writing – review & editing, Data curation, Conceptualization. **Muhammad Bilal Sadiq:** Writing – review & editing, Writing – original draft, Supervision, Methodology, Formal analysis, Conceptualization.

## Declaration of competing interest

The authors declare that they have no known competing financial interests or personal relationships that could have appeared to influence the work reported in this paper
